# Supplementary material for: Snapshot multidimensional photography through active optical mapping
Source: Nat Commun. 2020 Nov 5;11:5602. doi: 10.1038/s41467-020-19418-0 (PMC7645682; doi:10.1038/s41467-020-19418-0)
Supplement: Supplementary file 1 — Supplementary Information [file 41467_2020_19418_MOESM1_ESM.pdf]

[Supplementary Information]

Snapshot multidimensional photography through active optical mapping

Jongchan Park[1,2,4], Xiaohua Feng[1,2,4], Rongguang Liang[3], and Liang Gao[1,2,4*]

[1]Department of Electrical and Computer Engineering, University of Illinois at Urbana-Champaign, Urbana, IL, 61801, USA

[2]Beckman Institute for Advanced Science and Technology, University of Illinois at Urbana-Champaign, Urbana, IL, 61801, USA

[3]College of Optical Sciences, the University of Arizona, Tucson, AZ 85721, USA

[4]Current address: Department of Bioengineering, University of California, Los Angeles, CA 90095, USA

*Correspondence: Prof. Liang Gao, Tel: (217) 300-9930, Email: gaol@ucla.edu

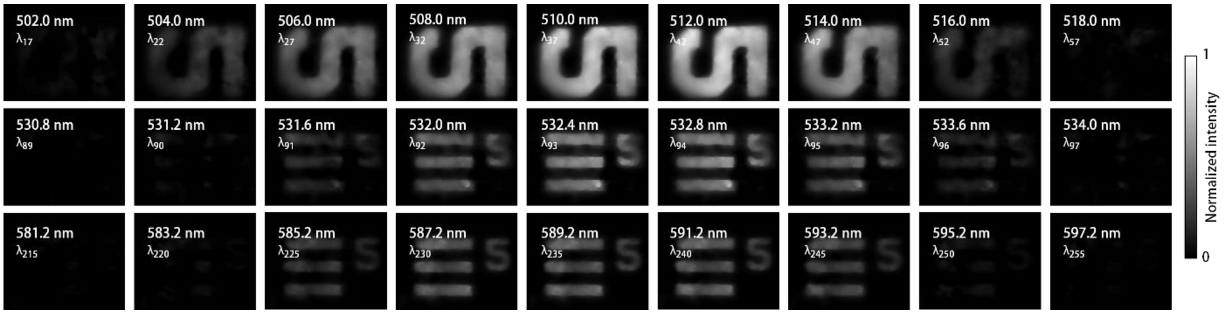

Supplementary Figure 1. (*x*, *y*, *λ*) imaging through compressed optical mapping. By using a compressed sensing method, a large-sized hyperspectral datacube was captured. The spectrum was sampled from wavelengths of 495.2 nm to 605.2 nm with a 0.4 nm step (a total of 275 samples in spectral domain). The spectral resolution was measured as 1.6 nm. Selected frames were shown in the figures.

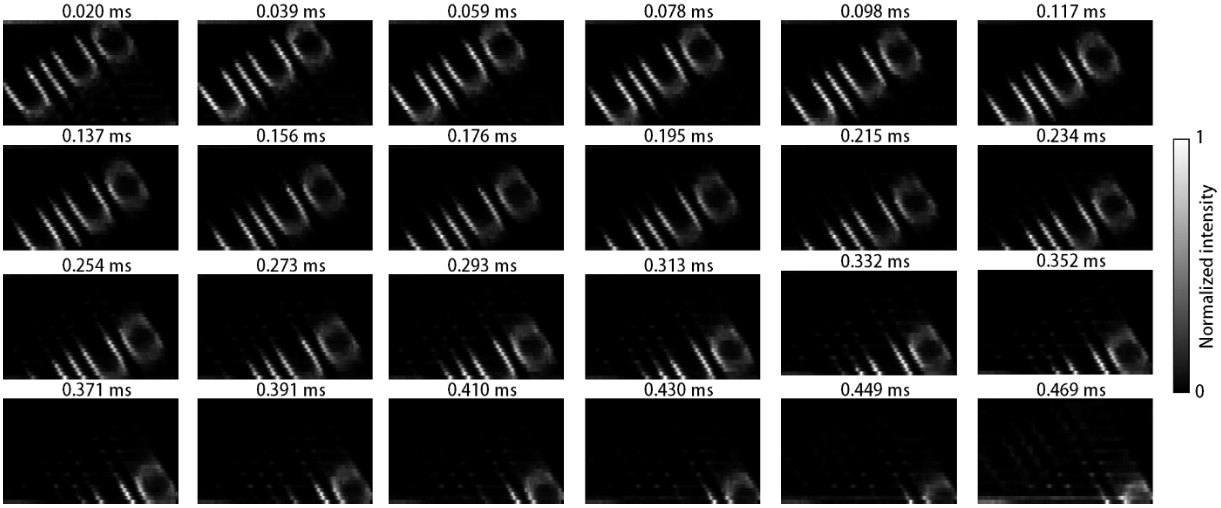

Supplementary Figure 2. (*x*, *y*, *t*) imaging through direct optical mapping. A fast-moving object was imaged by multidimensional photography at 51.2 kHz.

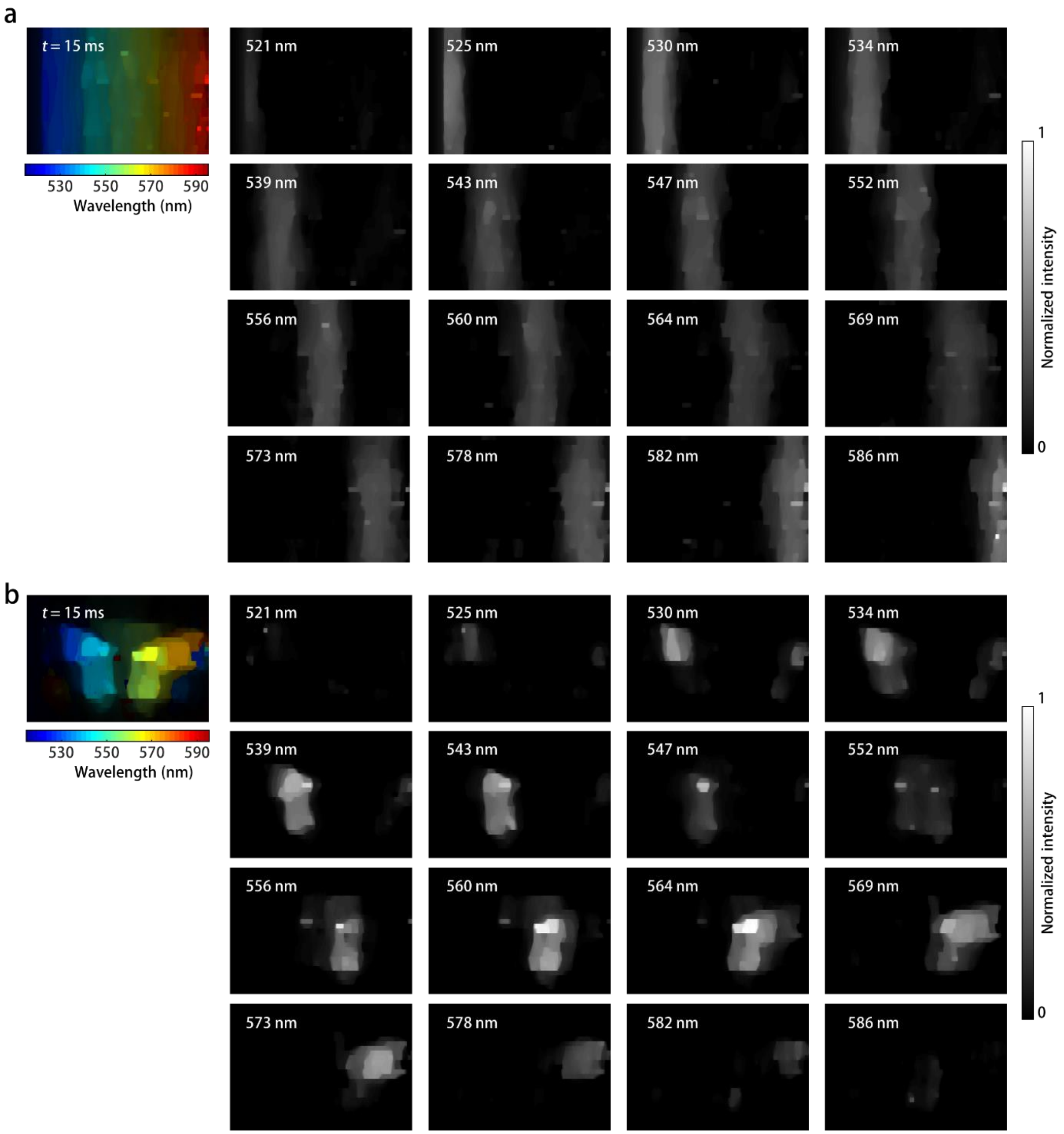

Supplementary Figure 3. (*x*, *y*, *λ*, *t*) imaging with hybrid optical mapping. Hyperspectral images of a linear variable filter (**a**) and the linear variable filter with a negative chrome mask (**b**) at a given time frame (t = 15 ms).

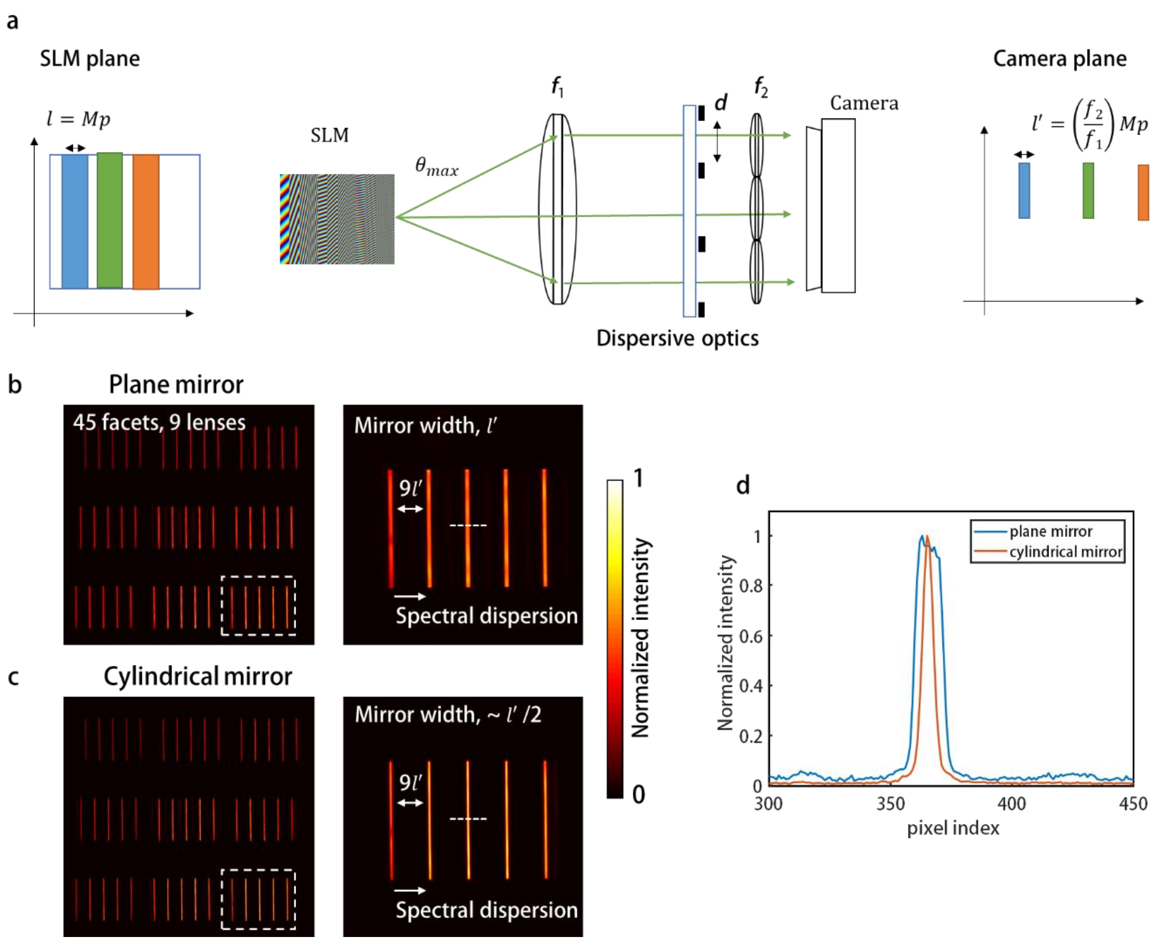

Supplementary Figure 4. Spectral resolution enhancement in hyperspectral imaging by using a cylindrical mapping pattern. a, Optical mapping with the SLM. The SLM vertically slices the incident image and remaps it onto the camera. Image slicing and mapping with (**b**) a plane pattern and (**c**) a cylindrical pattern. **d**, Intensity profiles of the image slices along the white dotted line in (**c**). The cylindrical pattern reduces the width of the image slice.

Supplementary Note 1. Spectral resolution enhancement in hyperspectral imaging with direct optical mapping

The spatial and spectral sampling rates of a conventional snapshot image mapping spectrometer are primarily dictated by the designs of the image mapper and relaying optics[1,2]. An incident image is vertically sliced by the image mapper consisting of tilted mirror facets, and mapped onto the camera through multiscale lenses[3]. The spatial sampling rate of the system along the horizontal direction is given by the number of mirror facets. The spectral sampling rate is given as a ratio between the distance between the sliced images along the dispersion axis and the width of the sliced image. For instance, an imaging spectrometer with 3×3 lens arrays, which vertically slices the images into 45 slices and remaps the images through 9 lenses, possesses a horizontal spatial sampling rate of 45 and a spectral sampling rate of 9 (Supplementary Figure 4b).

By contrast, the active mapper can increase the spectral resolution by imposing cylindrical or Fresnel lens patterns on the image slices. The patterns can reduce the width of the sliced image in the dispersion axis, thereby increasing the spectral resolution of the measurement. Because the width of the image slices converges upon propagation, we placed the detector at the focal plane of the cylindrical/Fresnel lens patterns.

Here, we derived the maximum achievable enhancement factor of the spectral resolution, η, in our system design and experimentally demonstrated it. Supplementary Figure 4a illustrates the concept of hyperspectral imaging using the SLM and the multiscale imager lenses. The incident image is vertically sliced and redirected by the SLM. The widths of the slices are *M* pixels. The slices are imaged onto the camera through lenses with focal lengths of f_1 and f_2. A dispersive element is placed at Fourier plane of the image to resolve the image along the spectral axis. A maximum deflection angle of the beam through the SLM is given as $\theta_m = \lambda/2p$ where *p* is a pixel pitch of the SLM and λ is a wavelength.

For simplicity, we only consider mapping of the images along horizontal directions at the SLM and the camera planes. With full utilization of addressable diffraction angle of the SLM, the propagating direction of the *N* image slices are equally distributed between 0° to θ_m. After the images pass through the front-end large collecting lens, the spatial separation of the sliced images at Fourier plane is

$$s = f_1 \frac{\theta_m}{N} = \frac{f_1 \lambda}{2Np}. \tag{1}$$

The slices are reimaged onto the camera through the lenslet array. Provided that the lenslet array limits the spatial resolution of the imaging system due to its small aperture, the size of the point-spread-function (PSF) of the system is given as

$$(\text{PSF}) = 1.22 \frac{\lambda}{NA} = 2.44 \frac{f_2 \lambda}{d}, \tag{2}$$

where *d* is a diameter of the pupils for the lenslet array. To avoid the crosstalk between the adjacent image slices, the spatial separation of the sliced images at the Fourier plane (or angular separations at the SLM plane) must be larger than, *d*.

$$\frac{f_1 \lambda}{2Np} = s > d = 2.44 \frac{f_2 \lambda}{(\text{PSF})}, \tag{3}$$

$$\frac{1}{2*2.44} \frac{1}{NP} \frac{f_1}{f_2} > \frac{1}{(\text{PSF})}. \tag{4}$$

The ratio between the width of the mirror slice at the camera plane and the size of the PSF is the maximum achievable spectral enhancement factor η, and it equals:

$$\eta = \frac{l'}{(\text{PSF})} = \frac{1}{2*2.44} \frac{1}{Np} \frac{f_1}{f_2} \cdot l', \tag{5}$$

$$\eta < \frac{1}{2*2.44} \frac{1}{Np} \frac{f_1}{f_2} \cdot \frac{f_2}{f_1} Mp, \tag{6}$$

$$\eta < 0.205 \frac{M}{N}. \tag{7}$$

In our experiment, a SLM with a horizontal resolution of 1920 pixels was used. An incident image was sliced into a total of 45 vertical slices. The images passed through 3 × 3 lens arrays, that five images share a lens. The width of each slice was 42 pixels. Therefore, M = 42, N = 3 (a number of imaging lenses at x-direction)

$$\eta < 2.8 \tag{8}$$

A maximum of 2.8-fold enhancement of the spectral resolution is expected. However, for experimental demonstrations, we utilise a portion of the maximum diffraction angle of the SLM due to low diffraction efficiencies at high angles. In addition, the imperfect performance of the plano-convex lenses of the lenslet array broaden the actual PSF. We experimentally demonstrated 2-fold enhancement of spectral resolution in our system (Supplementary Figure 4d). Although our demonstration in this section is limited to hyperspectral imaging (x, y, λ), the same argument applies to high-speed imaging (x, y, t).

In practice, the maximum spatial/spectral resolution of multidimensional photography is dictated by the optical design of the system and the performance of the SLM. Details are discussed in the following Supplementary Note 2.

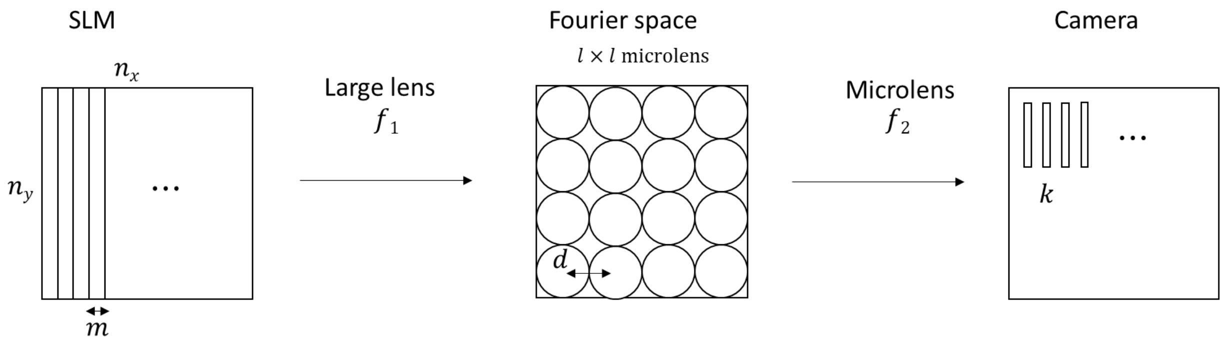

Supplementary Figure 5. Direct optical mapping with an SLM

Supplementary Note 2. Optical system designs and a space-bandwidth product of an SLM

Although multidimensional photography with an active optical mapper provides flexibility on measurement, achievable spatial and spectral/temporal sampling rates (the number of voxels of a light datacube) in the direct measurement scheme are fundamentally limited by the number of controllable degrees of freedom (i.e., pixel counts) of the SLM, which is also referred to as the space-bandwidth product (SBP) of the SLM.

Given an SLM with (n_x, n_y) pixels and a pixel pitch of p, an incident image is sliced and reflected by the SLM towards desired angles. To fully utilise the SBP of the SLM, we must choose angles that range across the maximum diffraction angle of the SLM, $\theta = \lambda/p$. Sliced images propagate through a lens with a focal length of f_1. A $l \times l$ microlens array captures the full Fourier spectrum of the SLM. Therefore, the relation between the diameter, d, of the each microlens and the number of microlenses is given as $d = f_1\,\lambda/pl$. Given m SLM pixels spanning the width of a mirror slice and k mirror slices imaged through the same microlens, the number of microlenses needed can be computed as $l \times l = n_x/mk$.

The spatial and spectral/temporal sampling rates of the light datacube are given as $N_x = n_x/m$, $N_y = n_y/m$, and $N_{\lambda,t} = n_x/mk$, respectively. The total number of voxels in the light datacubes is:

$$N_x \times N_y \times N_{\lambda,t} = n_x n_y \frac{n_x}{m^3 k} = N_{\mathrm{SLM}} \frac{n_x}{m^3 k} \tag{9}$$

where N_{SLM} is the SBP of the SLM. The microlens' PSF is given by:

$$\mathrm{PSF} = C\frac{f_2 \lambda}{d/2},\ C = 1.22 \text{ for Rayleigh criterion}\ . \tag{10}$$

Here f_2 is the focal length of the microlenses.

In our system, we match the mirror slice width at the camera plane, $w = mp\frac{f_2}{f_1}$, with the microlens' PSF:

$$mp\frac{f_2}{f_1} = \mathrm{C}\frac{f_2 \lambda}{d/2}. \tag{11}$$

Substituting d with $f_1\,\lambda/pl$ gives

$$m = 2Cl. \tag{12}$$

Further substituting l with $\sqrt{n_x/mk}$ leads to

$$\frac{n_x}{m^3 k} = \frac{1}{4C^2}. \tag{13}$$

Finally, we can then rewrite the total number of light field voxels as:

$$N_x \times N_y \times N_{\lambda,t} = N_{\text{SLM}} \frac{n_x}{m^3 k} = \frac{1}{4C^2} N_{SLM}. \tag{14}$$

Therefore, the voxel number of the light datacube is limited by the SBP of the SLM. To break this limitation, we can adopt a temporal-multiplexing strategy using multiple measurements. We show an example in Supplementary Figure 6.

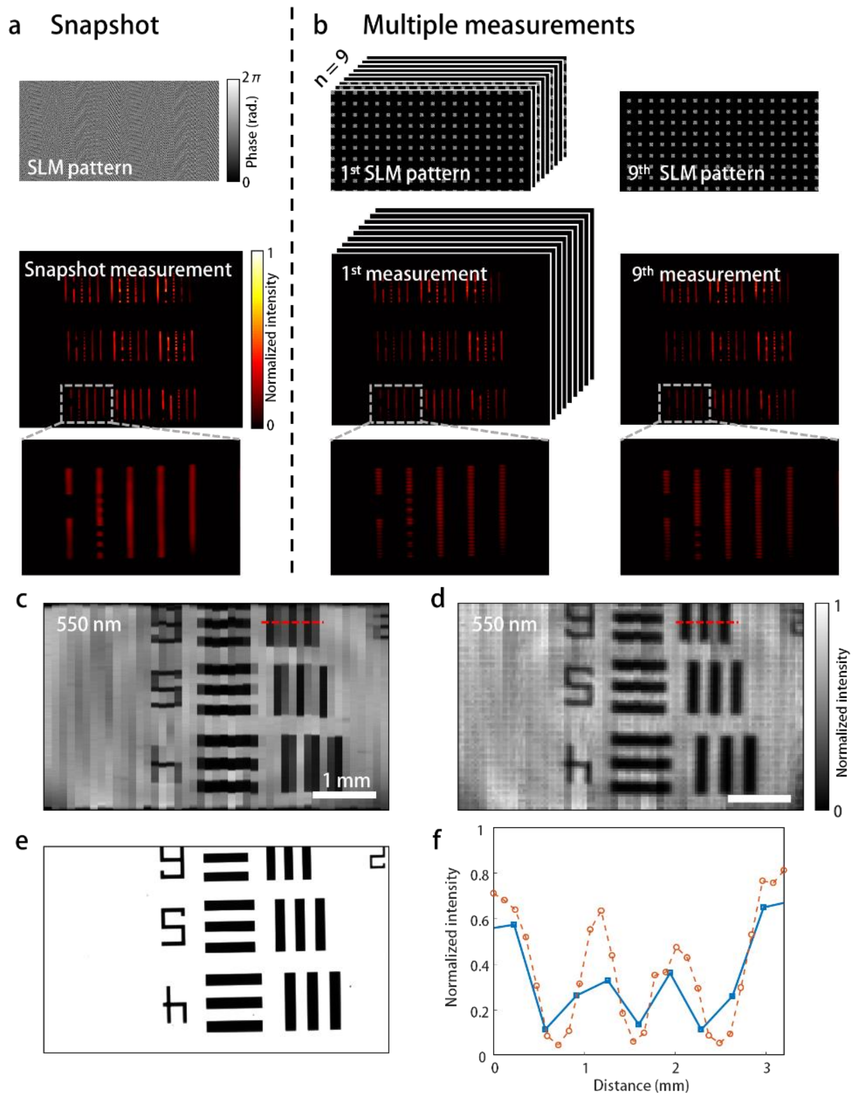

Supplementary Figure 6. Spatial resolution enhancement in direct optical mapping with multiple measurements. A 1951-USAF resolution target was illuminated with an incoherent beam (Central wavelength = 550 nm, Full width at half maximum = 10 nm) and served as an object. **a,** Snapshot measurement. The width of vertical mirror slices is 42 SLM pixels. **b,** Multiple measurements. The SLM pattern was divided into nine sub-patterns with equal spacing. Therefore, the width of slices in sub-patterns is 14 SLM pixels. One sub-pattern was displayed at a time, and a total of nine images were captured sequentially. Features of the incident image smaller than the width of the vertical slices in the snapshot measurement scheme (42 SLM pixels) were resolved. **c**, Image captured with the snapshot measurement at 550 nm. **d**, Image captured with the multiple measurements method at 550 nm. **e**, Ground-truth image captured with a monochromatic camera. **f**, Intensity profiles of the images in (**c**) and (**d**) along red dotted lines. The image from multiple measurements shows higher contrast with three times higher spatial sampling rates along the horizontal axis.

	Active-mapping snapshot multidimensional photography	IMS[1]	CASSI[4]	AFSS [5]	P2C2[6]	CACTI[7]	CUP[8]	STAMP[9]	Coded exposure photograph[10]
**Optical mapping mode**	Active	Passive	Passive	Passive	Passive	Passive	Passive	Passive	Passive
**Light datacube measured**	(x, y, λ), (x, y, t), or (x, y, λ, t)	(x, y, λ)	(x, y, λ)	(x, y, λ)	(x, y, t)	(x, y, t)	(x, y, t)	(x, y, t)	(x, y, t)
**Operating mode**	Direct, compressed, or hybrid	Direct	Compressed	Compressed	Compressed	Compressed	Compressed	Direct	Compressed

Supplementary Table 1. Comparison of active-mapping snapshot multidimensional photography against previous passive systems. IMS, image mapping spectrometer; CASSI, coded aperture snapshot spectral imager; AFSS, Adaptive Feature-Specific Spectrometer; P2C2, programmable pixel compressive camera; CACTI, coded aperture compressive temporal imaging; CUP, compressive ultrafast photography; STAMP, sequentially timed all-optical mapping photography.

Supplementary references

1. Gao, L., Kester, R. T., Hagen, N. & Tkaczyk, T. S. Snapshot Image Mapping Spectrometer (IMS) with high sampling density for hyperspectral microscopy. *Opt. Express* **18**, 14330–14344 (2010).
2. Bedard, N., Hagen, N. A., Gao, L. & Tkaczyk, T. S. Image mapping spectrometry: calibration and characterization. *Opt. Eng.* **51**, 111711 (2012).
3. Brady, D. J. & Hagen, N. Multiscale lens design. *Opt. Express* **17**, 10659–10674 (2009).
4. Wagadarikar, A., John, R., Willett, R. & Brady, D. Single disperser design for coded aperture snapshot spectral imaging. *Appl. Opt.* **47**, B44–B51 (2008).
5. Dunlop-Gray, M., Poon, P. K., Golish, D., Vera, E. & Gehm, M. E. Experimental demonstration of an adaptive architecture for direct spectral imaging classification. *Opt. Express* **24**, 18307–18321 (2016).
6. Reddy, D., Veeraraghavan, A. & Chellappa, R. P2C2: Programmable pixel compressive camera for high speed imaging. in *CVPR 2011* 329–336 (IEEE, 2011).
7. Llull, P. *et al.* Coded aperture compressive temporal imaging. *Opt. Express* **21**, 10526–10545 (2013).
8. Gao, L., Liang, J., Li, C. & Wang, L. V. Single-shot compressed ultrafast photography at one hundred billion frames per second. *Nature* **516**, 74–77 (2014).
9. Nakagawa, K. *et al.* Sequentially timed all-optical mapping photography (STAMP). *Nat. Photonics* **8**, 695–700 (2014).
10. Hitomi, Y., Gu, J., Gupta, M., Mitsunaga, T. & Nayar, S. K. Video from a single coded exposure photograph using a learned over-complete dictionary. in *2011 International Conference on Computer Vision* 287–294 (2011). doi:10.1109/ICCV.2011.6126254.
