# Supplementary material for: Snapshot multidimensional photography through active optical mapping
Source: Nat Commun. 2020 Nov 5;11:5602. doi: 10.1038/s41467-020-19418-0 (PMC7645682; doi:10.1038/s41467-020-19418-0)
Supplement: Supplementary file 3 — Description of Additional Supplementary Files [file 41467_2020_19418_MOESM3_ESM.docx]

**Description of Additional Supplementary Files:**

**Supplementary Movie 1.**

(*x*, *y*, *λ*) imaging with compressed optical mapping

**Supplementary Movie 2.**

Reconstruction of a 3D (*x*, *y*, *t*) datacube through iterative optimization

**Supplementary Movie 3.**

(*x*, *y*, *t*) imaging with compressed optical mapping

**Supplementary Movie 4.**

(*x*, *y*, *t*, *λ*) imaging with hybrid optical mapping
